# Immunomodulatory properties of CD38 antibodies and their effect on anticancer efficacy in multiple myeloma

**DOI:** 10.1002/cam4.6619

**Published:** 2023-10-15

**Authors:** Kamlesh Bisht, Taro Fukao, Marielle Chiron, Paul Richardson, Djordje Atanackovic, Eduardo Chini, Wee Joo Chng, Helgi Van De Velde, Fabio Malavasi

**Affiliations:** ^1^ Sanofi Oncology Cambridge Massachusetts USA; ^2^ Sanofi Research & Development Vitry‐sur‐Seine France; ^3^ Department of Medical Oncology, Jerome Lipper Multiple Myeloma Center Dana Farber Cancer Institute, Harvard Medical School Boston Massachusetts USA; ^4^ University of Maryland Marlene and Stewart Greenebaum Comprehensive Cancer Center Baltimore Maryland USA; ^5^ Department of Medicine University of Maryland School of Medicine Baltimore Maryland USA; ^6^ Department of Anesthesiology and Perioperative Medicine Mayo Clinic Jacksonville Florida USA; ^7^ Cancer Science Institute of Singapore National University of Singapore Singapore Singapore; ^8^ Department of Medical Sciences University of Turin Torino Italy; ^9^ Fondazione Ricerca Molinette Torino Italy

**Keywords:** adenosine, bone marrow niche, CD38 antibodies, daratumumab, immunomodulation, isatuximab, multiple myeloma

## Abstract

**Background:**

CD38 has been established as an important therapeutic target for multiple myeloma (MM), for which two CD38 antibodies are currently approved—daratumumab and isatuximab. CD38 is an ectoenzyme that degrades NAD and its precursors and is involved in the production of adenosine and other metabolites.

**Aim:**

Among the various mechanisms by which CD38 antibodies can induce MM cell death is immunomodulation, including multiple pathways for CD38‐mediated T‐cell activation. Patients who respond to anti‐CD38 targeting treatment experience more marked changes in T‐cell expansion, activity, and clonality than nonresponders.

**Implications:**

Resistance mechanisms that undermine the immunomodulatory effects of CD38‐targeting therapies can be tumor intrinsic, such as the downregulation of CD38 surface expression and expression of complement inhibitor proteins, and immune microenvironment‐related, such as changes to the natural killer (NK) cell numbers and function in the bone marrow niche. There are numerous strategies to overcome this resistance, which include identifying and targeting other therapeutic targets involved in, for example, adenosine production, the activation of NK cells or monocytes through immunomodulatory drugs and their combination with elotuzumab, or with bispecific T‐cell engagers.

## Graphical abstract

Daratumumab and isatuximab are two CD38 targeting antibodies approved for the treatment of multiple myeloma. In this review we summarize the biology of the CD38 target, various assets targeting CD38, mechanism of action of daratumumab and isatuximab and their immunomodulatory properties, resistance mechanisms and approaches to overcome resistance against CD38 antibodies. We also discuss the similarities and differences of the mechanism of action of these two antibodies, which might be important for immune modulation and overcoming immunosuppression in the myeloma bone marrow environment.

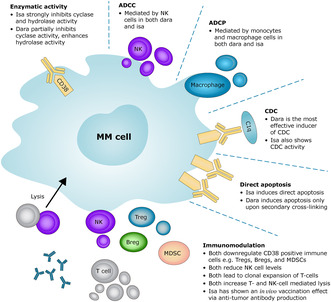

## 1 INTRODUCTION

Multiple myeloma (MM) is the second most common hematologic malignancy worldwide, characterized by malignant plasma cells (mPC) accumulating in the bone marrow (BM).^1^, ^2^ CD38 has been established as a key target for treating MM as both normal and malignant plasma cells have high levels of CD38 expression.^2^, ^3^, ^4^ In normal conditions, relatively low levels of CD38 are expressed on myeloid and lymphoid cells and non‐hematopoietic tissues, including T lymphocytes, NK cells, monocytes, and endothelial cells.^2^, ^3^, ^4^ Research has also shown that CD38 is also expressed on human platelet membranes, with nicotinamide adenine dinucleotide (NAD) glycohydrolase, and adenosine diphosphate (ADP) ribose cyclase, and therefore could possibly be involved in the signal transduction pathways that lead to platelet activation.^4^ The characterization of the CD38 protein and its overexpression on myeloma cells has resulted in the development of monoclonal antibodies (mAbs) targeting CD38, of which two antibodies—daratumumab and isatuximab—have been approved for clinical use.^2^, ^5^, ^6^, ^7^, ^8^, ^9^, ^10^

Daratumumab is approved for the treatment of newly diagnosed MM (NDMM) and relapsed/refractory MM (RRMM) in combinations that include proteasome inhibitors (PI) and immunomodulatory drugs (IMiDs).^11^, ^12^, ^13^, ^14^ Isatuximab is approved for the treatment of adult patients with RRMM in combination with pomalidomide and dexamethasone (Pd), and in combination with carfilzomib and dexamethasone (Kd), in patients who have received at least two prior therapies, including lenalidomide and a PI, or who have received one–three prior lines of therapy, respectively.^15^, ^16^, ^17^ Other CD38 antibodies currently under preclinical and clinical development for the treatment of MM include MOR202, TAK‐079, SAR442085, CID‐103, and FTL004.^6^, ^7^, ^9^, ^10^, ^18^

CD38 is also expressed in other hematologic malignancies, such as chronic lymphocytic leukemia, acute lymphocytic leukemia (ALL), acute myeloid leukemia, Waldenström's macroglobulinemia, and NK/T‐cell lymphoma.^19^, ^20^ The role of CD38 antibodies in treating these malignancies is being actively investigated. In preclinical studies, both daratumumab and isatuximab inhibited the growth of T‐ALL cell lines in vitro.^20^ One Phase 2 study has investigated daratumumab monotherapy for patients with relapsed/refractory NK/T‐cell lymphoma, with an overall response rate (ORR) of 25% but a short median duration of response.^21^ Case studies with daratumumab for the treatment of ALL have also been published, showing that some patients achieve minimal residual disease negativity (MRD−).^20^ There is also the potential to target CD38 in the treatment of cutaneous T‐cell lymphomas (CTCL), due to its dominant expression in some aggressive subtypes of CTCL.^22^ In patients with CTCL, expression levels of CD38, as detected by immunohistochemistry were also negatively correlated with overall survival.^22^, ^23^

CD38 also seems to play a role in the tumor microenvironments of solid tumors, with preclinical research revealing that CD38 is overexpressed in various tumor cell lines, such as nasopharyngeal carcinoma, hepatocellular carcinoma, and neuroblastoma cells.^24^ Clinical trials with CD38 mAbs have been conducted for the treatment of solid tumors such as glioblastoma, prostate cancer, non‐small cell lung cancer, and pancreatic cancer, although with limited efficacy data to continue future trials.^24^, ^25^, ^26^, ^27^, ^28^, ^29^

Apart from hematologic malignancies, case studies of daratumumab for the treatment of refractory systemic lupus erythematosus have also been published. In a study of two patients with refractory lupus who received daratumumab, both patients showed a decrease in disease activity, with downregulation of gene transcripts associated with T‐cell activation.^30^ CD38 antibodies are also being evaluated for kidney transplant rejection.^31^

This review will discuss the immunomodulatory properties of CD38 antibodies and their impact on MM treatment efficacy as well as mechanisms of therapeutic resistance.

## 2 CD38 PROTEIN

CD38 is a transmembrane glycoprotein that is expressed by normal T, B, NK, and myeloid cell populations and also in endothelial cells.^3^, ^32^ Expression of the *CD38* gene, present on chromosome 4, is regulated by a promoter region that contains binding sites for nuclear factor kappa B (NF‐κB), retinoid X receptor, liver X receptor, and signal transducer and activator of transcription (STAT).^33^ DNA demethylation is hypothesized to repress *CD38* expression, with the discovery of a CpG island on the first exon of *CD38*, and an inverse correlation between *CD38* promoter methylation status and the gene expression between normal and mPC in MM.^34^ Among immune subpopulations, NK cells, monocytes, and particularly M1 macrophages, have the highest cell surface CD38 receptor density, and CD38 is involved in the activation of these cells.^35^, ^36^, ^37^, ^38^, ^39^ As previously mentioned, CD38 is also expressed on platelets and may be involved in their activation.^4^ CD38 also binds to CD31 present on endothelial cells lining blood vessels, and inhibition of this interaction inhibits lymphocyte adhesion to endothelial cells.^40^ Comparisons between MM patients and normal BM donors have found that CD38 expression is higher on regulatory T cells (Tregs) than conventional T cells (Tcons), which allows CD38 mAbs to exert immunomodulatory effects through the reduction of Tregs.^41^, ^42^

**FIGURE 1:**
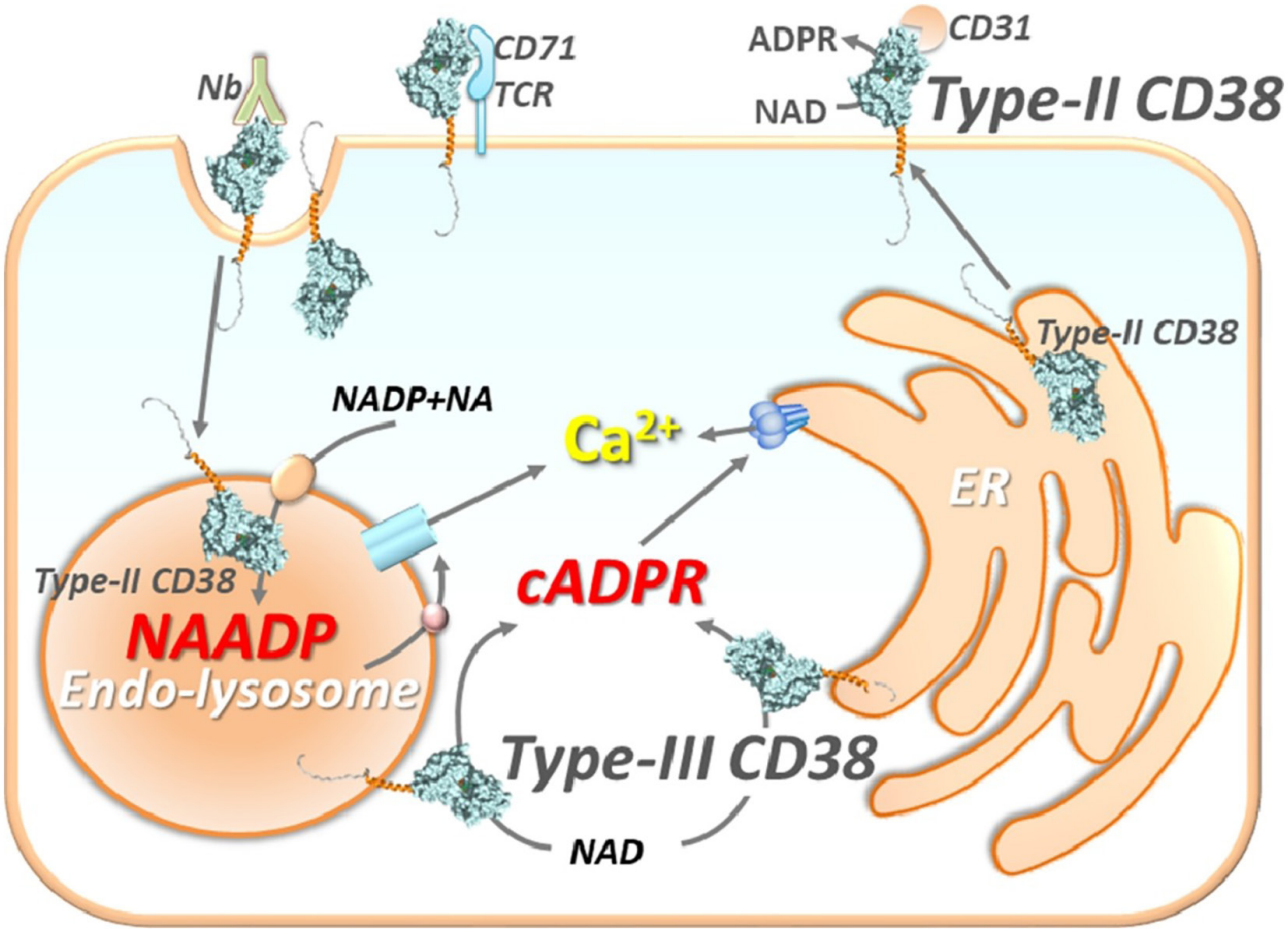
CD38 on the cell surface and in intracellular compartments, including endoplasmic reticulum. ADPR, adenosine diphosphate ribose; Ca, calcium; cADPR, cyclic adenosine diphosphate ribose; ER, endoplasmic reticulum; NAADP, nicotinic acid adenine dinucleotide phosphate; NAD, nicotinamide adenine dinucleotide; TCR, T‐cell receptor. Reprinted from Cell Calcium, Vol 101, Lee HC, Deng QW, Zhao YJ, The calcium signaling enzyme CD38—a paradigm for membrane topology defining distinct protein functions, 102514, Copyright (2022), with permission from Elsevier.

CD38 has multiple functions as a receptor and enzyme that is involved in nucleotide metabolism and calcium signaling. It is involved with various enzymatic activities that control NAD^+^ levels in the BM niche where the mPC grow.^32^ It is also implicated in heterotypic and homotypic cell adhesion.^3^, ^40^ CD38 is present on both the cell membrane and in intracellular compartments, including the mitochondria and endoplasmic reticulum (Figure 1).^43^ Depending on its orientation, the C‐terminal catalytic site may face the extracellular environment or be directed toward the cytosol, and the change in catalytic site from external to internal may be a mechanism to regulate its signaling activity.^43^, ^44^, ^45^ Activation of CD38 results in Ca^2+^ mobilization from the endoplasmic reticulum via ryanodine receptors, leading to an increase of intracellular Ca^2+^ concentration.^46^ Importantly, the majority of the CD38 enzyme exists as a Type II membrane protein with its catalytic site facing the outside of the cell, thus CD38 is mainly an ecto‐NAD glycohydrolase.^47^, ^48^

## 3 CD38 BIOLOGY IN THE MULTIPLE MYELOMA BONE MARROW NICHE

### 3.1 Canonical and noncanonical pathways of adenosine production

Extracellular adenosine (ADO) in the BM niche leads to immunosuppression and tumor growth.^32^ ADO is produced from the catabolism of ATP, NAD^+^, and cyclic adenosine monophosphate (cAMP). CD38 is involved in the noncanonical pathway of ADO production through the catabolism of NAD^+^ and NAD phosphate (NADP^+^).^49^, ^50^, ^51^, ^52^

**FIGURE 2:**
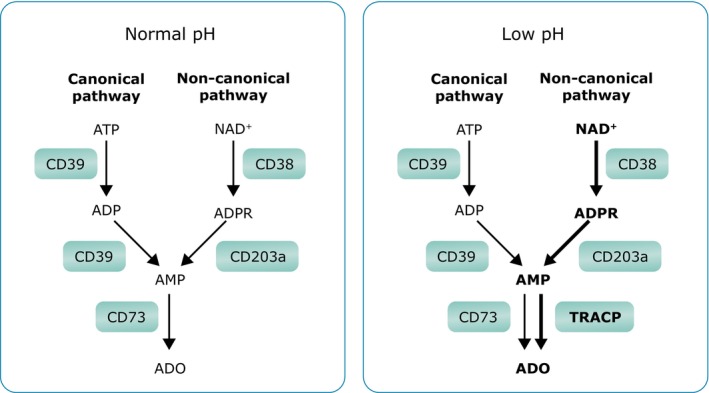
Canonical and noncanonical pathways of adenosine production in normal conditions and in the hypoxic MM BM niche. This figure shows the shift to noncanonical pathway of ADO production in the presence of a low pH environment, such as in the BM niche. An excess of NAD^+^ due to the shift from oxidative phosphorylation to aerobic glycolysis activates the CD38/CD203a/TRACP pathway, while the low pH also causes the inhibition of CD73. Additionally, ATP can be directly converted to AMP by CD203a. ADO, adenosine; ADP, adenosine diphosphate; ADPR, adenosine diphosphate ribose; AMP, adenosine monophosphate; ATP, adenosine triphosphate; CD203a, ectonucleotide pyrophosphatase/phosphodiesterase‐1; CD38, cyclic ADP‐ribose hydrolase; CD39, ectonucleoside triphosphate diphosphohydrolase‐1; CD73, 5′ nucleotidase; TRACP, tartrate‐resistant acid phosphatase.

In the canonical pathway of ADO production, ectonucleoside triphosphate diphosphohydrolase‐1 (CD39) converts ATP to ADP, and then to adenosine monophosphate (AMP).^32^, ^49^ In contrast, in the noncanonical pathway, CD38 metabolizes NAD^+^ to ADP‐ribose (ADPR), which is then converted to AMP by ectonucleotide pyrophosphatase/phosphodiesterase‐1 (CD203a/plasma cell‐1 [PC‐1]).^49^, ^50^, ^51^, ^52^ CD38 catalyzes the conversion of NAD^+^.^32^, ^53^, ^54^ Both the canonical and noncanonical pathways then converge to 5′‐nucleotidase (CD73), which fully degrades AMP to ADO (Figure 2).^49^ [Correction added on November 11, 2023 after first online publication. The ‘Figure 1’ has been changed to ‘Figure 2’ in the previous sentence.] An alternative PDE/CD73 pathway also converts cAMP to AMP to generate ADO.^51^

### 3.2 Metabolic reprogramming leads to an acidic, hypoxic BM niche that favors the noncanonical pathway

In the BM niche, ADO is primarily obtained from NAD^+^.^32^ This is due to metabolic reprogramming in the BM niche, which shifts from oxidative phosphorylation to aerobic glycolysis reducing ATP and simultaneously increasing NAD^+^. This excess of NAD^+^ activates the noncanonical pathway involving CD38 and CD203a to produce AMP.^50^ The shift to aerobic glycolysis also leads to an accumulation of lactic acid and a reduction in pH.^50^ A low pH induces a marked inhibition of ectonuclease CD73, and in the acidic and hypoxic conditions of the myeloma BM, the enzyme tartrate‐resistant acid phosphatase (TRACP) predominates in the conversion of AMP to ADO (Figure 2).^32^, ^50^, ^52^, ^55^

MM cells in the BM niche can also simultaneously express enzymes of the canonical or noncanonical ADO pathway, either on the surface of cells or on extracellular vesicles. These ectoenzymes may function according to continuous (molecules on the same cell) or discontinuous (molecules on different cells) spatial arrangements.^52^, ^56^ Inhibiting CD38 and CD73 might reduce total ADO production.^56^ Microvesicles from MM patients have also been found to express functional adenosinergic ectoenzymes that can produce ADO from ATP and NAD^+^,^57^ while treatment in vivo with daratumumab can induce the release of microvesicles expressing CD38, among other molecules such as CD39, CD73, CD203a, PD‐L1, CD55, and CD59.^58^ Release of microvesicles containing CD38 under isatuximab treatment has also been demonstrated, which was correlated with a decrease of CD38 expression on the plasma membrane in a MOLP8 cell line resistant to isatuximab.^59^ These findings support one possible mechanism of resistance against CD38 antibodies and will be discussed below in the “Mechanisms of Resistance Against CD38 Antibodies and Strategies” section.

In addition, the hypoxic BM niche induces the stabilization of hypoxia‐inducible factor 1‐alpha (HIF‐1α) in hematopoietic stem cells (HSCs),^60^ which increases the production of angiogenic factors and angiogenesis to increase oxygen delivery to tumor cells, and thereby stimulates tumor growth.^61^, ^62^, ^63^ This is crucial to support the survival and growth of MM cells in the initial stages of the disease—the mPC establish themselves within the endosteal niche of the BM, where they are exposed to greater levels of hypoxia and activating HIF‐1.^61^, ^62^, ^63^ Hypoxia also increases the expression of CD73 via HIF‐1, allowing for the accumulation of ADO from AMP in the tumor microenvironment that suppresses the immune response.^64^, ^65^ In murine MM models, inhibiting HIF‐1α downregulated pro‐angiogenic genes, including vascular endothelial growth factor, and led to a reduction in the weight and volume of the tumor burden.^66^

HIF‐1α is one part of the HIF heterodimeric complex, which is also composed of the HIF‐1β subunit.^67^ HIF‐1β is located in the 1q21 region of chromosome 1, and its expression is closely associated with gain (1q21), a common adverse chromosomal abnormality in MM.^67^ Hypoxia has also been shown to induce HIF‐1β expression in association with NF‐κB activation.^67^ Copy number gain is a prominent feature in myeloma and other cancers, and hypoxia‐induced 1q21 copy number gain or amplification (1q21+) has been reported in breast cancer cells.^68^ The contribution of the hypoxic BM environment in acquiring 1q21 copy number gain in myeloma is not known, but 1q21+ and hypoxic BM environment together can contribute to ADO production through both HIF‐1‐mediated CD73 upregulation,^67^, ^69^ and through the CD38/CD203a/TRACP pathway, and thus contribute to NK‐cell dysfunction in MM.^32^, ^50^, ^65^

### 3.3 ADO in the BM has an immunosuppressive effect, impacting T cells and NK cells

The accumulation of ADO in the BM niche modulates the communication between mPCs and normal cells, contributing to the immunocompromised state of MM patients.^70^ ADO impairs antitumor response through reducing CD8^+^ T‐cell cytotoxic functions and the T‐helper 1 CD4^+^ T‐cell response, while increasing the proportion of Treg cells and promoting myeloid‐derived suppressor cell (MDSC) expansion.^55^, ^71^ Tumor‐derived ADO can also markedly reduce NK‐cell‐mediated killing capacity, with the tumor microenvironment and exogenous ADO having been shown to enhance NK‐cell expression of CD73.^72^ Cytokine priming has also been observed to mitigate ADO‐induced immunosuppressive effects—ADO inhibits tumor necrosis factor alpha release from interleukin (IL)‐2 stimulated NK cells, and IL‐2 has been found to alleviate ADO‐induced immunosuppression of NK cells.^73^ Evidence from solid tumors with a selective agonist of the adenosine A_2A_ receptor (ADORA2A) demonstrates tumors contain immunosuppressive concentrations of ADO that inhibit antitumor immune responses in numerous ways. The mechanisms that may be inhibited by ADO include the activation, differentiation, and clonal expansion of tumor‐specific T cells with helper and cytotoxic effector functions, as well as cytotoxic T cells (CTL) binding to syngeneic tumor cells, and tumor cell destruction by NK cells, lymphokine‐activated killer cells, and CTL. The extracellular ADO produced by the tumor inhibits antitumor T cells after triggering elevation of intracellular levels of cAMP by ADORA2A, which suppresses T‐cell receptor signaling and interferon (IFN)‐γ production.^74^

ADO ligation with ADORA2A leads to decreased proliferation and inhibition of the cytolytic antitumor activities of CTL, and inhibition of cytotoxicity and IFN‐γ release by NK cells. These effects lead to an increased number of immunoregulatory cells, such as Tregs, establishing an immunosuppressive environment.^32^, ^75^

Furthermore, MDSCs can accumulate in the BM microenvironment in the initial stages of tumor development and mediate MM progression through suppressing T‐cell activation and inducing MM cell survival.^76^, ^77^ Preexisting CD8^+^ T‐cell responses against myeloma‐associated antigens have been detected in MM patients.^78^

## 4 THERAPEUTIC APPLICATIONS OF ANTI‐CD38 TARGETING ANTIBODIES

**FIGURE 3:**
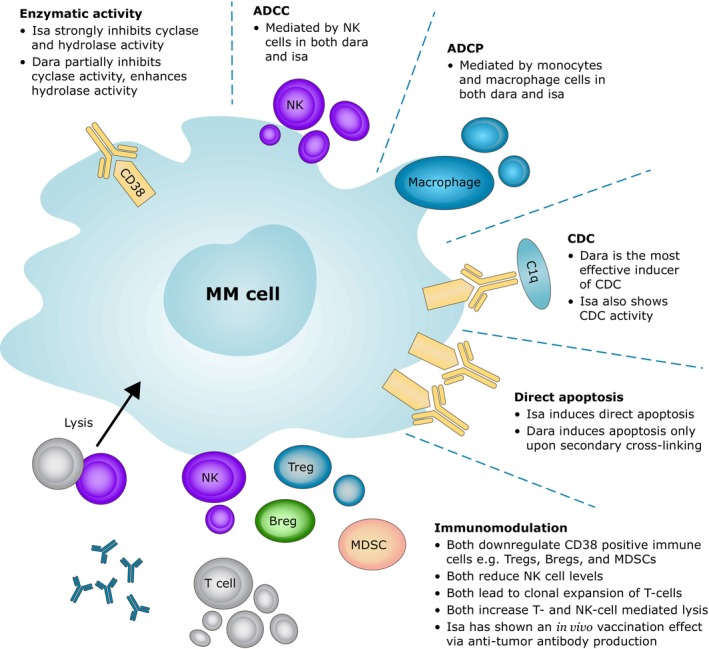
Different mechanisms of action of anti‐CD38 antibodies. ADCC, antibody‐dependent cellular cytotoxicity; ADCP, antibody‐dependent cellular phagocytosis; Breg, regulatory B cell; CDC, complement‐dependent cytotoxicity; Dara, daratumumab; Isa, isatuximab; MDSC, myeloid‐derived suppressor cell; NK, natural killer; Treg, regulatory T‐cell.

CD38 antibodies kill tumor cells through a number of mechanisms—antibody‐dependent cellular cytotoxicity (ADCC), antibody‐dependent cellular phagocytosis (ADCP), complement‐dependent cytotoxicity (CDC), direct apoptosis, enzymatic inhibition, and immunomodulation (Figure 3).^42^ The two currently approved CD38 antibodies, daratumumab and isatuximab, have some key differences in the mechanisms of action.

Daratumumab and isatuximab bind to different, nonoverlapping epitopes on the CD38 molecule, with isatuximab binding to a specific epitope that partially encompasses the CD38 ectoenzyme catalytic site without blocking its access or altering configuration.^79^ Daratumumab, in contrast, has a binding site located outside of the CD38 catalytic site.^79^

### 4.1 ADCC, ADCP, CDC, and direct apoptosis

ADCC‐mediated MM cell death occurs via NK‐cell degranulation and the release of perforins and granzymes.^80^, ^81^ Lysis of MM cells treated with CD38 mAb is dose‐dependent and can occur in the presence of bone marrow‐derived mesenchymal stem/stromal cells, and in patient‐derived peripheral blood mononuclear cells (PBMCs).^81^ ADCC is the main mechanism of isatuximab‐induced tumor cell death, as was demonstrated in a study of tumor cell depletion in vitro in primary tumor plasma cells from 13 patients.^2^, ^82^ However, during treatment, the contribution of ADCC to the overall activity of CD38 mAbs decreases over time due to their induction of NK‐cell fratricide.^83^, ^84^, ^85^, ^86^ In most patients treated with daratumumab, PBMCs isolated during treatment can still induce some level of ADCC, showing that the remaining group of NK cells maintains cytotoxic functionality.^81^ A new anti‐CD38 mAb, FTL004, has also exhibited enhanced proapoptotic activity and displayed stronger ADCC against CD38^+^ malignant cells but has not been evaluated in clinical trials as yet.^10^

In ADCP, binding with Fc gamma receptors (FcγRs), present on monocytes and macrophages, induces phagocytosis of antibody‐opsonized tumor cells.^42^ Phagocytosis contributes to the antitumor activity of the CD38 antibodies isatuximab and daratumumab.^42^

CDC occurs through the binding of the CD38 anti‐CD38 mAb complex with the complement‐activating protein C1q. The C1q binds to the Fc domain of the antibody, initiating the complement cascade.^42^, ^80^, ^81^ Direct cell lysis occurs when the terminal complement pathway generates membrane attack complexes that build pores in the MM cell membrane.^42^, ^80^, ^81^ Of the CD38 mAbs, daratumumab is the most effective inducer of CDC, having been shown to induce high levels of CDC at a low concentration in the BM microenvironment.^81^ Daratumumab was originally selected from a panel of 42 CD38‐specific mAbs based on potent CDC activity^87^ and its efficacy has been shown to be negatively impacted by higher expression of complement inhibitory proteins CD55 and CD59, of which the former is found on chromosome 1q.^87^, ^88^, ^89^, ^90^, ^126^ [Correction added on November 11, 2023 after first online publication. The reference 126 has been included in the previous sentence.]

Direct apoptosis is mediated through the activation of caspases‐7 and ‐8, lysosome permeabilization, and upregulation of reactive oxygen species.^42^, ^81^, ^82^, ^91^ Currently, isatuximab is the only available CD38 antibody capable of inducing direct apoptosis; daratumumab, MOR202 and TAK‐079 induce apoptosis upon secondary cross‐linking.^2^, ^54^, ^79^, ^80^ The apoptotic activity of isatuximab was observed in MOLP‐8 MM cell lines, which express high levels of CD38.^2^ An in vitro study of CD38^+^ MM tumor cell lines demonstrated that daratumumab‐mediated apoptosis occurs after cross‐linking by cells expressing either activating FcγR or the inhibitory FcγRIIb.^81^, ^92^ In primary cells from BM aspirates of MM cells, isatuximab‐induced apoptosis without any addition of external cross‐linking agents and independent of effector cells.^93^, ^94^ Experiments with the Fab′ fragment of isatuximab and daratumumab as part of a conjugate with morpholino oligonucleotide (MORF1) also demonstrated the Fab′ fragment of isatuximab‐induced levels of apoptosis comparable to that of the whole antibody, in contrast to the daratumumab Fab′ fragment which showed little apoptotic efficacy.^95^

### 4.2 Impacts on enzymatic activity

Both daratumumab and isatuximab modulate the enzymatic activity of CD38 in vitro.^2^, ^32^, ^79^, ^80^ Isatuximab inhibits both CD38 hydrolase and cyclase activity, while daratumumab only partially inhibits cyclase activity and enhances hydrolase activity.^2^, ^19^, ^53^ Ectoenzyme activity assays showed that isatuximab inhibited 70% of cyclic guanosine diphosphate ribose (cGDPR) generation as compared with 20% with daratumumab, both at 30 μg/mL.^96^ cGDPR generation is indicative of the combined CD38 cyclase activity that generates fluorescent cGDPR.^96^ Inhibition of CD38 ectoenzymatic activity is a unique feature of isatuximab, whereas daratumumab has nearly no direct inhibition of CD38 enzymatic activity.^2^, ^18^, ^97^This might be due to the unique binding mode of isatuximab as, although significant conformational changes occur in CD38 upon isatuximab binding, the catalytic site remains. As previously mentioned, isatuximab and daratumumab bind to different, nonoverlapping epitopes of CD38.^79^ Thus, isatuximab is likely to inhibit CD38 enzymatic activity through allosteric inhibition.^93^ Other anti‐CD38 antibodies show much less or no potent inhibition of CD38 enzymatic activity.^2^, ^93^

### 4.3 Anti‐CD38 immunomodulatory effects

CD38 antibodies also produce immunomodulatory effects in the BM niche.^98^ Daratumumab exerts immunomodulatory effects via the elimination of CD38^+^ immune suppressor cells, such as Tregs, regulatory B cells, and MDSCs.^42^ Exposure to daratumumab also leads to higher levels of granzyme B in T cells, indicating improved killing capacity.^42^ T‐cell clonality and functional antiviral responses, as measured by IFN‐γ production, also increased with daratumumab treatment, indicating that T cells continue to function properly despite low CD38 expression.^99^ Increased T‐cell response may thus be due to a depletion of regulatory cells.^99^ These changes in T‐cell expansion, activity, and clonality were more prominent in daratumumab responders than nonresponders.^99^ Deep immune profiling of whole‐blood samples from RRMM patients in the Phase 3 POLLUX study at baseline and after 2 months of treatment (lenalidomide/dexamethasone [Rd] or daratumumab plus Rd [D‐Rd]) showed a reduced NK‐cell population and persistence of a phenotypically distinct NK‐cell population characterized by increased expression of HLA‐DR, CD69, CD127, and CD27. Deep responders to D‐Rd showed a higher proportion of CD8^+^ versus CD4^+^ T cells, an increased proportion of T cells, especially effector memory cells, and a reduction in Tregs. The study revealed clonal expansion of CD8^+^ T cells, suggesting the generation of an adaptive immune response in the daratumumab arm.^100^

Another study also using high‐dimensional mass cytometry of whole‐blood and BM samples from RRMM patients treated with daratumumab monotherapy revealed decreased concentrations of NK cells, B cells, and CD38^+^ Tregs after 2 months of treatment.^86^ The study also found that the NK‐cell population exhibited an increase in CD69 and CD127 expression, and in patients who responded to therapy, there was a shift to a CD8^+^ T‐cell population upon treatment, with significant increases in granzyme B production.^86^

A study exposing MM BM cells to daratumumab also revealed a CD14^+^ CD16^+^ monocyte subset that mediated MM cell killing. Using a mAb to inhibit the CD47/signal‐regulatory protein alpha (SIRPα) axis, which is found on monocytes, also increased daratumumab‐mediated cell death.^101^

Isatuximab also inhibits the suppressive function of Tregs by reducing their numbers, blocking their trafficking, and decreasing immune inhibitor cytokine production including IL‐10.^42^ This results in improved NK‐ and T‐cell mediated antitumor immune responses.^42^ Isatuximab monotherapy also induced expansion of clonotypic T cells, demonstrated by an increase in T‐cell receptor (TCR) clonality in the peripheral blood over the first three treatment cycles.^85^ The addition of dexamethasone to isatuximab was not associated with any further increase in TCR clonality.^85^ Independent of its ectoenzyme activity, CD38 is also a co‐stimulatory molecule for T‐cell activation and isatuximab functions as an agonist to drive T‐cell activation.^102^

Isatuximab has been shown to preferentially block Tregs compared with Tcons owing to the increased CD38 levels on Tregs in PBMCs.^41^ The same study also found that CD38 expression on Tregs is also upregulated by the IMiDs lenalidomide and pomalidomide.^41^ Isatuximab was shown to decrease the proliferation of Tregs in a dose‐dependent manner, which was enhanced by pomalidomide more than lenalidomide.^41^ Isatuximab also upregulated CD8^+^ T‐ and NK‐cell‐mediated lysis of MM cells, which was also enhanced more by pomalidomide than lenalidomide.^41^

Moreno et al. have also revealed that when cocultured with MM cells, isatuximab induces NK‐lymphocyte activation beyond tumor‐NK cell cross talk, through increased expression of CD69 and CD137 after Fc binding and CD38 transmembrane signaling.^82^ They also observed that in primary BM samples from MM patients, isatuximab significantly depleted tumor plasma cells, CD38^hi^ B‐lymphocyte precursors, basophils, and NK lymphocytes.^82^ The depletion of NK lymphocytes seemed to be due to activation followed by exhaustion after isatuximab treatment, and not by fratricide alone.^82^

Immune monitoring after treatment with isatuximab has revealed an overall decrease in total NK cells in both responders and nonresponders to isatuximab. However, CD38^+^ NK cell levels seem to remain stable in patients who respond to treatment, in particular, the CD38^+^ CD56^+dim^ and CD16^+bright^ cytotoxic subset.^85^ Separately, NK cells with CD38 knockout were found to be resistant to isatuximab‐induced fratricide, as has been demonstrated similarly with daratumumab.^84^, ^103^ Treatment with daratumumab leads to reduced NK cell levels due to fratricide, followed by recovery posttreatment.^104^

An in vivo vaccination effect with isatuximab has also been observed in MM patients; in a small study of four patients, those with preexisting anti‐myeloma immune responses were more likely to develop antitumor immune responses under isatuximab treatment.^105^ Two patients had very few or no preexisting antibody responses, and through treatment with isatuximab, did not develop any new serological antitumor immune responses or objective clinical responses. In contrast, the other two patients with preexisting antibody responses against numerous myeloma‐associated antigens, showed continuous increases in their antitumor antibody titers during isatuximab treatment.^105^ These two patients achieved complete remission after the first and third cycle of isatuximab.^105^ Tumor‐specific immune fitness may be associated with clinical responses to mAb treatment in myeloma patients.^105^

Another hypothesis could be that daratumumab achieves partial ectoenzyme inhibition of CD38, whereas isatuximab can cause complete inhibition of CD38 ectoenzyme activity.^2^, ^19^, ^32^, ^53^, ^79^, ^80^ This strong ectoenzyme inhibition makes isatuximab more effective in overcoming ADO‐mediated immunosuppression in the MM hypoxic tumor microenvironment, which promotes ADO generation predominantly through the CD38/CD203a/TRACP pathway. Further studies are needed to test this hypothesis but may explain clinical observations of greater isatuximab activity, such as is seen in the treatment of extramedullary MM.^106^, ^107^

### 4.4 Clinical trial findings for CD38 antibodies in patients with MM

The immunomodulatory effects of the different CD38 antibodies are likely to contribute to the clinical antitumor activities of these agents, although their exact role would be hard to identify within a set of mechanisms of action. CD38 antibodies have been combined with PIs and IMiDs for NDMM patients eligible and ineligible for autologous stem cell transplantation, and for RRMM patients.^43^ Table 1 summarizes the efficacy analyses of various anti‐CD38 treatment strategies in randomized Phase 3 clinical trials in both the NDMM and RRMM settings.

**TABLE 1:** Summary of available Phase 3 clinical trial efficacy results for anti‐CD38 treatments in MM.

Trial name	Disease setting	Treatment arms	Key results
*Newly diagnosed MM*			
CASSIOPEIA^108^	Transplant‐eligible	Dara‐VTd vs. VTd	sCR: 29% vs. 20% ≥CR: 39% vs. 26% MRD−: 9.2% vs. 5.4% at end of induction; 33.7% vs. 19.9% following consolidation mPFS: NR vs. NR (HR: 0.47; 95% CI: 0.33–0.67; *p* < 0.0001)
GMMG‐HD7^109^	Transplant‐eligible	Isa‐RVd vs. RVd	MRD−: 50% vs. 36% at end of induction CR: 24% vs. 22% ≥VGPR: 77% vs. 61%
MAIA^110^	Transplant‐ineligible	Dara‐Rd vs. Rd	mPFS: NR vs. 34.4 mo (HR: 0.53; 95% CI: 0.43–0.66; *p* < 0.0001) mOS: NR vs. NR (HR: 0.68; 95% CI: 0.53–0.86; *p* = 0.0013)
ALCYONE^111^	Transplant‐ineligible	Dara‐VMP vs. VMP	PFS: HR: 0.42; 95% CI: 0.34–0.51 OS: HR 0.60; 95% CI: 0.46–0.80; *p* = 0.0003
*RRMM*			
APOLLO^112^	RRMM with ≥1 previous line of therapy	Dara‐Pd vs. Pd	mPFS: 12.4 mo vs. 6.9 mo (HR: 0.63; 95% CI: 0.47–0.85; *p* = 0.0018) ORR: 69% vs. 46% ≥CR: 25% vs. 4% ≥VGPR: 51% vs. 20% MRD−: 9% vs. 2%
POLLUX^113^, ^114^	RRMM with ≥1 prior line of therapy	Dara‐Rd vs. Rd	mPFS: 44.5 mo vs. 17.5 mo (HR: 0.44; 95% CI: 0.35–0.55; *p* < 0.0001) mOS: 67.6 mo vs. 51.8 mo (HR: 0.73; 95% CI: 0.58–0.91; *p* = 0.0044) ORR: 93% vs. 76% ≥CR: 57% vs. 23% MRD−: 30% vs. 5%
CASTOR^115^	RRMM with ≥1 previous line of therapy	Dara‐Vd vs. Vd	mPFS: 16.7 mo vs. 7.1 mo (HR: 0.31; 95% CI: 0.25–0.40; *p* < 0.0001) ORR: 85% vs. 63% ≥CR: 30% vs. 10% ≥VGPR: 63% vs. 29% MRD−: 14% vs. 2%
CANDOR^116^, ^117^	RRMM with 1–3 previous lines of treatment	Dara‐Kd vs. Kd	mPFS: 28.6 mo vs. 15.2 mo (HR: 0.59; 95% CI: 0.45–0.78; *p* < 0.0001) ORR: 84% vs. 75% CR: 29% vs. 10% MRD−: 18% vs. 4%
IKEMA^118^	RRMM with 1–3 previous lines of therapy	Isa‐Kd vs. Kd	mPFS: 35.7 mo vs. 19.2 mo (HR: 0.58; 95.4% CI: 0.42–0.79) ORR: 87% vs. 84% CR: 44% vs. 29% MRD−: 34% vs. 15%
ICARIA‐MM^119^, ^120^	RRMM with ≥2 previous lines of treatment	Isa‐Pd vs. Pd	mPFS: 11.5 mo vs. 6.5 mo (HR: 0.596; 95% CI: 0.44–0.81; *p* = 0.001) mOS: 24.6 mo vs. 17.7 mo (HR: 0.76; 95% CI: 0.57–1.01; *p* = 0.028) ORR: 60% vs. 35% ≥VGPR: 32% vs. 9%

Abbreviations: CI, confidence interval; CR, complete response; d, dexamethasone; Dara, daratumumab; HR, hazard ratio; Isa, isatuximab; K, carfilzomib; M, melphalan; mo, month; MM, multiple myeloma; mOS, median overall survival; mPFS, median progression‐free survival; MRD−, minimal residual disease negativity; NR, not reached; ORR, overall response rate; OS, overall survival; P, pomalidomide; PFS, progression‐free survival; R, lenalidomide; RRMM, relapsed/refractory MM; sCR, stringent complete response; T, thalidomide; V, bortezomib; VGPR, very good partial response.

Both isatuximab and daratumumab have been shown to improve the clinical outcome in high‐risk MM patients. A meta‐analysis of 2340 daratumumab‐treated patients from CASSIOPEIA, MAIA, and ALCYONE (for NDMM), and CASTOR and POLLUX (for RRMM) showed that the daratumumab‐based regimens led to a progression‐free survival (PFS) benefit in standard‐risk NDMM (hazard ratio [HR] 0.43; 95% CI: 0.35–0.53; *p* < 0.05) and in both standard‐risk and high‐risk RRMM patients (HR 0.28; 95% CI: 0.21–0.36; *p* < 0.05 and HR 0.48; 95% CI: 0.30–0.76; *p* < 0.05, respectively).^121^ Another meta‐analysis of daratumumab clinical trials that included the CANDOR trial for RRMM concluded that addition of daratumumab led to improved PFS in patients with standard‐risk NDMM (pooled HR 0.45; 95% CI: 0.37–0.54; *p* < 0.001) and high‐risk NDMM (pooled HR 0.67; 95% CI: 0.47–0.95; *p* = 0.02), as well as standard‐risk RRMM (pooled HR 0.38; 95% CI: 0.26–0.56; *p* < 0.001) and high‐risk RRMM (pooled HR 0.45; 95% CI: 0.30–0.67; *p* < 0.001) patients.^122^ Both meta‐analyses defined high‐risk cytogenetics as the presence of *t*(4;14), *t*(14;16), or del(17p) and none of these studies included data on 1q21+ patients.^121^, ^122^

Subgroup analysis published from Phase 3 IKEMA study demonstrated that the addition of isatuximab to Kd resulted in improved PFS in both RRMM patients with standard‐risk (HR 0.44; 95% CI: 0.27–0.73) and high‐risk cytogenetics (HR 0.72; 95% CI: 0.36–1.45).^123^ A similar subgroup analysis from the ICARIA‐MM Phase 3 trial showed the benefit of isatuximab in high‐risk [del(17p), *t*(4;14), or *t*(14;16)] patients.^124^ The addition of isatuximab to pomalidomide–dexamethasone led to longer median PFS (mPFS) (7.5 vs. 3.7 months; HR, 0.66; 95% CI: 0.33–1.28) in high‐risk patients.^124^ This analysis also showed benefit of Isa‐Pd in the 1q21 subgroup.^124^

Copy number gain of 1q21 is one of the most common chromosomal abnormalities in MM, with its incidence increasing with disease progression.^125^, ^126^ Analysis of BM microenvironment samples from GMMG‐HD7 trial patients by flow cytometry and single‐cell RNA sequencing also revealed that patients with 1q21+ and *t*(4;14) had significantly increased numbers of monocytes.^127^ Hyperdiploid patients with 1q+ also showed significant depletion of B‐cell lineage cell types.^127^ Some recent reports have analyzed outcomes in patients with 1q21+ receiving different anti‐CD38 therapies, with the results showing possible differences in outcomes among the different regimens evaluated.^128^, ^129^, ^130^ In the MAIA trial investigating daratumumab plus lenalidomide and dexamethasone (Dara‐Rd) in transplant‐ineligible NDMM patients, subgroup analysis of PFS in patients with 1q21+ and more than 1 other high‐risk chromosomal abnormality showed an HR of 1.03 (95% CI: 0.42–2.48).^131^ A subgroup analysis of the GRIFFIN trial investigating daratumumab in combination with bortezomib, lenalidomide, and dexamethasone (Dara‐RVd) in transplant‐eligible NDMM patients reported a PFS HR of 0.81 (95% CI: 0.15–4.47), in favor of the Dara‐RVd combination, in patients with 1q21+ and another high‐risk chromosomal abnormality.^132^ However, the percentage of patients with 1q21+ was small and no cut‐off values for affected cell ratios were provided.^132^ A real‐world analysis in Sweden of NDMM patients found the presence of amp(1q) was independently associated with a shorter OS.^133^

In RRMM, subgroup analyses of patients with 1q21+ have been reported for the ICARIA‐MM and IKEMA studies investigating isatuximab.^128^ In ICARIA‐MM, the mPFS in patients with 1q21+ was 9.5 months versus 3.8 months in the Isa‐Pd versus Pd arms, respectively (HR = 0.40; 95% CI: 0.25–0.63).^128^ In IKEMA, mPFS in patients with 1q21+ was not reached and 16.2 months, respectively, in the Isa‐Kd and Kd arms (HR = 0.57; 95% CI: 0.33–0.98).^128^ A larger percentage of patients with isolated 1q21+ achieved MRD− with Isa‐Kd (36.2%) versus Kd alone (9.7%); these were similar to the proportions in standard‐risk patients (36.9% vs. 14.0%).^128^

A retrospective analysis of RRMM patients with 1q21+ treated with daratumumab‐based regimens (monotherapy, Dara‐Rd, Dara‐Pd, or Dara‐Vd) was recently presented.^129^ In this analysis, 57 of 278 patients (20%) had 1q21+, and gain1q21 was present in a majority of patients; only 4 patients had amp1q21.^129^ The mPFS for 1q21+ patients treated with daratumumab‐based regimens was 24.6 months, and ORR was 57.9%.^129^ No mPFS difference was observed for patients with 1q21+ versus patients without 1q21+ (24.5 months vs. 23.5 months, respectively).^129^ In contrast, a small real‐world retrospective study noted a suboptimal outcome in 8 consecutive daratumumab‐treated RRMM patients who had amp1q21, with an mPFS of 3.0 months after a median follow‐up of 10.01 months.^134^ Only 1 patient achieved very good partial response, and 7 patients discontinued treatment, all due to disease progression.^134^ In the same period, in RRMM patients without amp1q21 (*n* = 40), mPFS was not reached after a median follow‐up of 18.4 months.^134^ A real‐world analysis of the Czech Registry of Monoclonal Gammopathies of Dara‐Rd found no statistically significant benefit to Dara‐Rd in patients with gain(1q21) versus patients who received Rd, with a hazard ratio of 1.33 for PFS by univariable Cox model.^135^ Another study analyzed the prognostic impact of 1q21+ and gene expression profiling of 70 genes (GEP70) risk score in 81 RRMM patients treated with daratumumab‐based regimens.^130^ Patients with 1q21+ showed a median PFS and median OS of 0.5 years and 0.9 years, respectively, whereas the median PFS for non‐1q21+ patients was 2.1 years and median OS was not reached.^130^

Further investigation into the different outcomes of patients with 1q21+ receiving daratumumab or isatuximab is ongoing. A possible mechanism that could contribute to the difference in outcome could be the overexpression of complement regulatory proteins CD46, CD55, and CD59 in the 1q21+ subgroup. [Correction added on November 11, 2023 after first online publication. The reference 88 has been removed from the previous sentence.] The complement inhibitor proteins CD55 and CD59 have been implicated in daratumumab resistance.^88^, ^130^ Interestingly, the *CD55* gene is located on chromosome 1q32.2. Gains of chromosome 1q can also occur at a whole‐arm‐level in patients with 1q21+, which can lead to a mild upregulation of other genes located on 1q, such as the previously mentioned HIF‐1β.^67^, ^126^, ^136^ [Correction added on November 11, 2023 after first online publication. The references 67 and 126 are included in the previous sentence.]

## 5 MECHANISMS OF RESISTANCE AGAINST CD38 ANTIBODIES AND POTENTIAL STRATEGIES TO OVERCOME RESISTANCE

**FIGURE 4:**
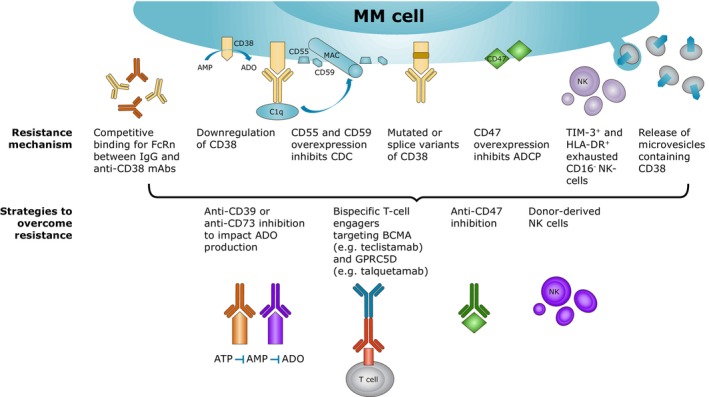
Summary of resistance mechanisms and strategies to overcome resistance. ADO, adenosine; BCMA, B‐cell maturation antigen; FcRn, neonatal Fc receptor; IgG, immunoglobulin G; NK, natural killer.

Mechanisms of resistance against CD38 antibodies can be divided into tumor intrinsic resistance mechanisms, and immune microenvironment‐related resistance mechanisms, and numerous strategies are currently being investigated to overcome these resistance mechanisms (Figure 4).

### 5.1 Tumor intrinsic resistance mechanisms

A multitude of tumor resistance mechanisms have been described in the literature that include resistance to ADCP, CDC, as well as impacts to CD38 expression and CD38 protein. Recent insights have suggested that daratumumab resistance may not be solely explained by the complete loss of CD38 or loss of binding of daratumumab to MM cell surface CD38. Instead, Viola et al. have proposed that daratumumab resistance might be attributable to a combination of downregulation of CD38 and upregulation of factors including toll‐like receptor 8, CD47, and the chemokines CXCL10 and CXCL4 on immune microenvironment cells.^137^

Downregulation of CD38 expression has been observed with daratumumab. Nijhof et al. reported that in a cohort of 102 patients treated with daratumumab monotherapy (16 mg/kg), CD38 expression was reduced in both BM‐localized and circulating MM cells after the first daratumumab infusion.^88^ After discontinuation of daratumumab, CD38 expression levels on MM cells increased again.^88^ Low levels of CD38 expression on myeloma cells during daratumumab treatment are hypothesized to be due to trogocytosis of the CD38‐daratumumab complex by monocytes and granulocytes, whereby a cell extracts and ingests “bites” of material from another cell.^138^, ^139^, ^140^

The overexpression of complement regulatory proteins such as CD55 and CD59 is another mechanism that helps MM cells resist CDC. As previously mentioned, CD55 overexpression has been suggested to contribute to daratumumab resistance in patients harboring 1q21+.^130^ [Correction added on November 11, 2023 after first online publication. The reference 88 has been replaced with 130 in the previous sentence.] Nijhof et al. showed a significant increase in CD55 and CD59 expression in patients who developed progressive disease during daratumumab monotherapy.^88^ Lymphoma models have also demonstrated a negative impact of CD55 and CD59 on daratumumab activity, which may be a prevalent resistance mechanism. Daratumumab typically induces marginal CDC in mantle cell lymphoma, follicular lymphoma, or chronic lymphocytic leukemia cells.^89^, ^90^ This limited CDC induction may be explained by the high expression of complement inhibitors CD46, CD55, and CD59, and by insufficient expression of CD38.^141^

Resistance to ADCP may also occur through the overexpression on MM cells of CD47, which binds to SIRPα on tumor‐associated macrophages (TAMs) and contributes to the immune escape of tumor cells by inhibiting ADCP.^142^ Daratumumab has been combined with a CD47 antibody in a preclinical model of pediatric T‐cell ALL, and was able to prolong survival of the mice compared with single‐agent treatments.^101^, ^143^

Another potential mechanism that may contribute to CD38 antibody resistance is competitive binding to the neonatal FcR (FcRn), which is a cell‐surface molecule that protects IgG proteins from degradation during recycling.^144^, ^145^ Endogenous disease‐produced IgG, in particular the IgG type M‐protein, may compete with CD38 mAbs for FcRn, potentially leading to a shorter half‐life for the therapeutic IgG, although this has not been shown clinically.^145^

Mutations may also impact CD38 and its ability to react with CD38 antibodies.^146^ In a recent paper by Vo et al., integrative clinical sequencing of 511 RRMM patients revealed mutations that impacted the NF‐κB and RAS/mitogen‐activated protein kinase signaling pathways, including frameshift mutations that abolished CD38 expression.^146^ The authors also discovered a point mutation that affected codons 221–250, and disrupted most of the CD38 epitopes bound by daratumumab, thus facilitating the evasion of MM cells from daratumumab.^146^ Theoretically, the mechanism of action of isatuximab would not be affected by this point mutation, as the epitope of isatuximab is composed of residues from codons 34–189.^146^ No mutations in CD38 have been reported to impact isatuximab binding.

Adamia and colleagues have identified alternate splice variants of CD38 that abrogate the binding sites for anti‐CD38 antibodies.^147^ Four splice variants were found from total and single‐cell RNA sequencing of six samples from MM patients treated with daratumumab or isatuximab in combination with other MM drugs.^147^ The splice variants encoded functional proteins and were overexpressed in a daratumumab‐resistant cell line, and protein folding analysis revealed that these splice variants evaded the binding by daratumumab and isatuximab.^147^

A genome‐wide CRISPR‐Cas9 knockout screen by Liu et al. found that the *KDM6A* gene regulates CD38 and CD48 expression in MM. Further investigations revealed that restoration of CD48, downregulated by the knockout of *KDM6A*, restored NK‐cell activity and led to resensitization of cells to daratumumab treatment. *KDM6A* encodes a lysine‐specific demethylase that demethylates lysine 27 of histone H3 (H3K27) to promote gene expression. Their research found that an EZH2 inhibitor, which methylates H3K27, upregulated both CD38 mRNA and protein expression in *KDM6A* knockout cells, and restored the sensitivity of these knockout cells to daratumumab‐mediated NK‐cell cytotoxicity in vitro and in xenograft mouse models.^148^

### 5.2 Immune microenvironment‐related resistance mechanisms

The previously mentioned effect of ADO on NK cells also contributes to resistance mechanisms. The ligation of ADO to ADORA2A leads to decreased proliferation of cytotoxic T lymphocytes and their inhibition of cytolytic antitumor activities, as well as inhibition of cytotoxicity and IFN‐γ release by NK cells, resulting in a long‐lasting immunosuppressive environment.^32^

Evidence suggests that changes to NK cell numbers and function also occur over the MM disease course.^149^, ^150^ The number of peripheral blood NK cells seems to decrease during the progression of monoclonal gammopathy of undetermined significance (MGUS) to early‐stage untreated MM,^151^ and to reduced numbers in advanced‐stage untreated patients.^152^ NK cells may also lose their cytotoxic capacity in advanced MM.^152^ In MM, transforming growth factor beta produced by plasma cells, Tregs, or potentially MDSCs, has been reported to downregulate NK‐activating receptors and impair NK‐cell functions.^152^ A recent report showed NK‐cell dysfunction plays a major role in primary and acquired resistance to daratumumab. A reduced proportion of CD16^+^ and granzyme B^+^ NK cells and higher frequency of TIM‐3^+^ and HLA‐DR^+^ exhausted‐NK cells was present at baseline in patients resistant to daratumumab treatment. The presence of exhausted NK cells was an independent predictor for inferior PFS and OS with daratumumab treatment.^153^

Differences among patients in their BM NK cell composition have also been found to determine the response to daratumumab.^154^ A recent study by Tahri et al. reported flow cytometric and single‐cell RNA sequencing analyses of BM NK‐cell composition in 19 NDMM patients. Four patients (~20%) had a reduced frequency of cytotoxic CD56^dim^ NK cells together with an altered BM NK transcriptome that featured enriched expression of genes encoding enrichment of inhibitory receptors (*KLRB1* and *KLRC1*) and decreased expression of genes encoding activating receptors such as *FCGR3A* (CD16), *NCR3* (NKp30), and *CD226* (DNAM‐1). In this subgroup, the relative reduction in cytotoxic NK‐cell levels correlated with a significantly shorter PFS following daratumumab‐based therapy.^154^

As mentioned above, NK cells also express relatively high levels of CD38.^84^ Research has shown both daratumumab and isatuximab treatment may induce a depletion of peripheral blood and BM NK cells via fratricide ADCC against nearby NK cells.^84^, ^85^ Another hypothesis for the decrease of NK cells during daratumumab treatment is that daratumumab induces NK cell‐exhaustion via degranulation and IFN‐γ release from only the CD38^+^ NK‐cell population, which reduces their populations, and leaves an activated CD38^−^ NK‐cell population.^155^

Recruitment of monocytes via cytokines produced by the tumor microenvironment also leads to the development of TAMs that promote the survival of myeloma cells, as well as monocyte‐derived MDSCs that have immunosuppressive effects.^156^, ^157^, ^158^ Monocytes are recruited to the tumors through the expression of CCR2, the receptor for CCL2, a monocyte chemoattractant cytokine produced by the tumor microenvironment.^156^ Macrophages promote the growth of and decrease apoptosis of myeloma cell lines in cultures.^156^ Notably, plasmacytomas, which occur in MM, are also rich in TAMs.^156^

Yu et al. have demonstrated that *PHF19*, an epigenetic gene correlated with poor prognosis in MM patients, mediates the immunosuppressive BM microenvironment by promoting induced Treg frequency, and decreasing CD38 expression on MM cells.^159^ Cells overexpressing *PHF19* were less susceptible to in vitro NK‐mediated lysis induced by daratumumab or isatuximab, with diminished daratumumab‐induced cytotoxicity in mice models.^159^

Research has also shown that the binding of daratumumab to CD38 modifies cytoskeleton reorganization in MM cells by inducing a redistribution of CD38 antigens into polar aggregates that are released into the BM microenvironment. Isatuximab has also demonstrated release of microvesicles containing CD38, which correlates with a decrease of CD38 expression on the plasma membrane of MM cells.^58^, ^59^ As mentioned above, these aggregates can act both locally within the BM and at a distance via the bloodstream and may be considered one mechanism of anti‐CD38 mAb resistance.^57^, ^160^, ^161^ These microvesicles express CD38 and other ectoenzymes such as CD73, leading to decreased CD38 expression. These microvesicles can also produce ADO from ATP and NAD^+^.^51^, ^82^ This contributes to the increase of ADO levels in the BM niche, generating immune suppression via the modulation of pro‐ and anti‐inflammatory cytokine release.

### 5.3 Strategies to overcome resistance

An already utilized strategy to overcome resistance or limited response, is the combination of CD38 mAbs with IMiDs. IMiDs are able to induce NK‐cell activation, and lenalidomide and pomalidomide also upregulate CD38 on MM cells, resulting in the synergistic enhancement of the cytotoxic effects of CD38 antibodies.^42^, ^162^, ^163^, ^164^, ^165^ IMiDs have also demonstrated improvements in daratumumab‐mediated ADCC in lenalidomide‐refractory MM cells, while pomalidomide enhances isatuximab‐induced ADCC in vitro and in vivo.^2^, ^42^ As discussed previously, isatuximab has been shown to directly induce MM cell death via lysosomal‐associated and apoptotic pathways, and this activity is further enhanced in combination with pomalidomide.^94^ MOR202 also synergizes with pomalidomide via multiple mechanisms including direct cytotoxicity, and CD38 upregulation.^164^ Combining MOR202 with lenalidomide also led to enhanced ADCP activity.^166^ The combination of anti‐CD38 targeting therapies with IMiDs can also combat the decrease in CD38^high^ NK cells that is observed with CD38 mAbs.^81^ Taken together, these observations show that CD38 antibodies and IMiDs work synergistically through a combined effect of multiple mechanisms, which include direct cytotoxicity, CD38 upregulation, and effector cell activation. Elotuzumab (anti‐SLAMF7) is another therapeutic anti‐myeloma antibody that has been shown to enhance NK‐cell activation and is currently being evaluated in a Phase 2 trial of daratumumab‐refractory patients.^167^, ^168^

Currently, CD38 antibodies are also combined with PIs such as carfilzomib and bortezomib, however the exact mechanisms by which these combinations exert their efficacy are less clear than combinations with IMiDs.^42^ A flow cytometry‐based assay has shown bortezomib enhances the therapeutic efficacy of daratumumab through the sensitization of tumor cells for antibody‐mediated lysis.^169^ Daratumumab has also been shown to internalize CD38 from the MM cell surface and impair MM cell adhesion to stromal cells, the latter of which sensitizes MM cells to bortezomib‐induced killing.^170^ Daratumumab and isatuximab have been combined with PIs, showing clinical benefit as detailed previously in Table 1.^116^, ^117^, ^118^

One of the strategies to address resistance to CD38 antibodies typically involves inhibiting molecules overexpressed in MM or molecules involved in ADO production.

For instance, as CD39 is also involved in the production of ADO and is expressed on the cell surface of tumor cells in human cancer cell lines including myeloma, CD39 could be a therapeutic target in overcoming resistance to current therapies for treating MM.^171^, ^172^ CD39 expression can be transcriptionally induced and upregulated on endothelial cells, along with the upregulation of CD73 expression.^171^ Inhibition of CD73 could also overcome immune suppression and restore lysis of MM cells by autologous T cells.^173^ Plasmacytoid dendritic cells (pDCs) interacting with MM cells have been shown to induce CD73 in both pDCs and MM cells, and anti‐CD73 mAb treatment of a pDC‐MM co‐culture model decreased ADO levels and triggered cytotoxic T lymphocyte activity against autologous MM cells.^70^ A Phase 1 clinical trial of a potent, selective, orally bioavailable CD73 inhibitor, ORIC‐533 is currently underway to examine the use of CD73 inhibition to improve outcomes in patients with RRMM.^173^, ^174^

Targeting CD39 and CD73 in combination could be a strategy to reduce levels of ADO that drive the immunosuppressive environment, as inhibition at multiple nodes is likely to produce an additive or synergistic effect. Inhibition of two enzymes in the canonical pathway of CD39 and CD73 with IPH5201 and IPH5301, respectively, showed a synergistic effect and more effective inhibition of the ADO pathway.^175^ IPH5201, the CD39‐targeting antibody, is currently being investigated in a Phase 1 study in combination with durvalumab (anti‐PD‐L1) with or without oleclumab (CD73 mAb) in adult patients with advanced solid tumors.^176^, ^177^ Combining CD38 and CD73 inhibition synergistically could be a strategy to overcome resistance from the microvesicles released after treating with an anti‐CD38 agent, particularly since the CD38 pathway is more active in MM. However, there are no current trials investigating the combination of CD38 and CD73 inhibition.

Another potential therapeutic target is CD47, a cell‐surface ligand overexpressed in hematological malignancies, including NDMM.^178^ Gene expression of *CD47* directly correlates to the stage of disease, with plasma cells from MM patients overexpressing CD47 compared with MGUS patients.^179^ CD47 is also upregulated in drug‐resistant MM cells. miR‐155, a microRNA, has been found to directly target CD47 and is downregulated in drug‐resistant MM cells.^180^ Restoration of miR‐155 in drug‐resistant MM cells induced phagocytosis of MM cells by macrophages.^180^ Blocking CD47 with a CD47 mAb has been shown to increase phagocytosis of myeloma cells in vitro, and retard the growth of patient myeloma cells and alleviate bone resorption in human bone‐bearing mice.^181^ A number of CD47 monoclonal and bispecific antibodies are being investigated in Phase 1 and 2 trials for the treatment of MM and other solid tumors.^142^ ISB 1442 is one such bispecific antibody, targeting CD38 and CD47, and is currently being investigated in a Phase 1/2 study for RRMM patients.^182^

Upregulation of CD38 by all‐trans retinoic acid (ATRA) has been proposed as a strategy to potentiate the effect of anti‐CD38 antibodies. ATRA was shown to decrease expression of CD55 and CD59, enhancing the effect of daratumumab in vitro and in a mouse model.^165^ However, combining ATRA with daratumumab did not provide a marked benefit in a Phase 1/2 study of patients with RRMM.^183^ Inhibition of the JAK/STAT3 pathway has also been shown to produce a similar upregulating effect on CD38 expression, and ruxolitinib suppressed STAT3 activation and enhanced daratumumab‐mediated ADCC in MM cell lines.^53^

T‐cell engagers are also emerging as a complementary strategy when combined with an anti‐CD38 therapy, as they target a desired MM antigen and the CD3 subunit of the TCR.^184^ Teclistamab is a recently approved bispecific antibody targeting B‐cell maturation antigen (BCMA) and CD3 that was investigated in the MajesTEC‐1 study for RRMM patients, while talquetamab is a bispecific antibody targeted against CD3 and G protein‐coupled receptor family C group 5 member D (GPRC5D).^185^, ^186^ Subcutaneous teclistamab and talquetamab are being investigated in combination with daratumumab for RRMM in a Phase 1b study, TRIMM‐2, with the rationale that it might improve efficacy.^187^ Initial results showed that the combination of teclistamab and daratumumab had preliminary efficacy in pretreated patients with MM, while talquetamab and daratumumab showed deep and durable responses in heavily pretreated patients with RRMM.^187^, ^188^ ISB 1342, a bispecific T‐cell engager antibody binding to a different CD38 epitope is currently in Phase 1 studies, and has been shown to elicit potent antitumor activity in samples from RRMM patients who have previously received daratumumab.^189^ A tri‐specific T‐cell engager antibody, SAR442257, has also been developed that simultaneously binds to CD3, CD28, and CD38, and is in Phase 1 investigation.^190^, ^191^ The recognition of CD38 by SAR442257 would redirect T‐cells against MM.^190^, ^191^ SAR442257 showed a high response rate in samples from MM patients who relapsed after anti‐CD38 and anti‐BCMA therapy.^192^ A number of other T‐cell engagers are also being developed, including elranatamab and cevostamab.^193^, ^194^

## 6 CONCLUSION

CD38 is an ectoenzyme that plays a role in the noncanonical pathway of ADO production in the BM microenvironment. Its high and uniform expression on MM and plasma cells still makes it an attractive target for the treatment of MM.

In addition to the other mechanisms of action that result in tumor cell death or phagocytosis, CD38 has immunomodulatory effects on the BM tumor microenvironment, with multiple pathways for CD38‐mediated T‐cell activation. Targeting CD38 with CD38 mAbs such as daratumumab and isatuximab therefore also leads to immunomodulatory effects, such as the suppression of Tregs and the engagement of NK cells and macrophages. Resistance mechanisms to CD38 antibodies include tumor intrinsic mechanisms such as the downregulation of CD38 expression and alternative splicing of CD38 to prevent binding with antibodies, as well as immune microenvironment resistance mechanisms that include the change in NK‐cell phenotype over the course of the disease.

Strategies to overcome resistance are being investigated, including targeting other ectoenzymes in the adenosinergic pathway, such as CD73 and CD39. These strategies lead to multiple opportunities for promising combinations, including simultaneous targeting of CD38 and CD47. Future trials could investigate the synergistic effect of CD38 with CD73 or CD47 inhibition. Moreover, the ability to combine CD38 mAbs with other agents and their consistent translation in efficacy from clinical trials to real‐world practice continues to provide a therapeutic platform for improving patient outcome.^195^

## AUTHOR CONTRIBUTIONS

**Kamlesh Bisht:** Conceptualization (equal); writing – original draft (equal); writing – review and editing (equal). **Taro Fukao:** Conceptualization (equal); writing – review and editing (equal). **Marielle Chiron:** Writing – original draft (equal); writing – review and editing (equal). **Paul Richardson:** Writing – review and editing (equal). **Djordje Atanackovic:** Writing – review and editing (equal). **Eduardo Chini:** Writing – review and editing (equal). **Wee‐Joo Chng:** Writing – original draft (equal); writing – review and editing (equal). **Helgi van de Velde:** Conceptualization (equal); writing – review and editing (equal). **Fabio Malavasi:** Writing – original draft (equal); writing – review and editing (equal).

## CONFLICT OF INTEREST STATEMENT

**KB, TF, MC**, and **HVdV** are employed by Sanofi and may hold stock and/or stock options in the company. **PR:** Grants and funding—BMS/Celgene, Karyopharm, Oncopeptides, Takeda; Consulting—AstraZeneca, BMS/Celgene, GSK, Karyopharm, Oncopeptides, Protocol Intelligence, Regeneron, Sanofi, Secura Bio, Takeda; **WJC**: Grants and funding—Sanofi; Honoraria—AbbVie, BMS, GSK, Janssen, Pfizer, Sanofi, Takeda; Meeting support—Janssen, Sanofi; **DA**: Research support—Sanofi. **FM** has nothing to disclose.

## Data Availability

Data sharing is not applicable to this article as no new data were created or analyzed in this study.
